# Nematode Symbiont for *Photorhabdus asymbiotica*

**DOI:** 10.3201/eid1210.060464

**Published:** 2006-10

**Authors:** John G. Gerrard, Susan A. Joyce, David J. Clarke, Richard H. ffrench-Constant, Graeme R. Nimmo, David F.M. Looke, Edward J. Feil, Lucy Pearce, Nick R. Waterfield

**Affiliations:** * Gold Coast Hospital, Southport, Queensland, Australia;; † University of Bath, Bath, United Kingdom;; ‡ University of Exeter in Cornwall, Falmouth, United Kingdom;; § Queensland Health Pathology Service, Brisbane, Queensland, Australia;; ¶ Princess Alexandra Hospital, Brisbane, Queensland, Australia

**Keywords:** *Photorhabdus*, nematodes, insects, dispatch

## Abstract

*Photorhabdus asymbiotica* is an emerging bacterial pathogen that causes locally invasive soft tissue and disseminated bacteremic infections in the United States and Australia. Although the source of infection was previously unknown, we report that the bacterium is found in a symbiotic association with an insect-pathogenic soil nematode of the genus *Heterorhabditis*.

Most newly recognized human pathogens are zoonotic (i.e., able to infect nonhuman animal species) (*1*). Although it is well established that vertebrates are associated with emerging human infectious disease, the role of invertebrates, which constitute >95% of known animal species, has received far less attention.

The genomics era, however, has resulted in a dawning recognition of the importance of invertebrates in the emergence of human infection (*2*). For example, the virulent insect pathogen *Bacillus thuringiensis* is genetically closely related to the human pathogen *Bacillus anthracis*, the cause of anthrax (*3*). *Yersinia pestis*, the cause of plague, contains insecticidal toxins, which may have been laterally transferred from the insect pathogen *Photorhabdus luminescens* (*4*).

*Photorhabdus* organisms are γ-proteobacteria that display the curious property of bioluminescence (they glow in the dark); 3 species are currently recognized: *P*. *asymbiotica*, *P*. *luminescens*, and *P*. *temperata* (*5*). The latter 2 species have been intensively studied by entomologists because they are virulent insect pathogens. They form a symbiotic relationship with nematodes (*Heterorhabditis* sp.) that invade the larvae of insects. The nematodes regurgitate the bacteria, which kill the insects and provide a food source for the nematodes. Insect-pathogenic nematodes are thought to be harmless to vertebrates and are used in horticulture for biologic control of insects (*6*).

*P*. *asymbiotica* is a human pathogen, the source of which has not previously been identified. First described in 1989 by Farmer et al. (*7*), *P*. *asymbiotica* has been associated with invasive soft tissue and disseminated bacteremic infections in the United States and Australia. Multifocal skin and soft tissue abscesses are characteristic. Reported predominantly from Texas and the eastern coast of Australia, *P*. *asymbiotica* infections have been associated with outdoor activity during the warm summer months (*8*). Because this bacterium was not believed to be associated with nematodes, it was given the name *asymbiotica* (not a symbiont) in 1999 (*5*).

The organism can be isolated from soft tissue or blood samples and grows readily on conventional bacterial culture media. However, because clinical microbiology laboratories may misidentify *P*. *asymbiotica*, the true frequency of human infection is uncertain (*9*).

## The Study

**Figure 1:**
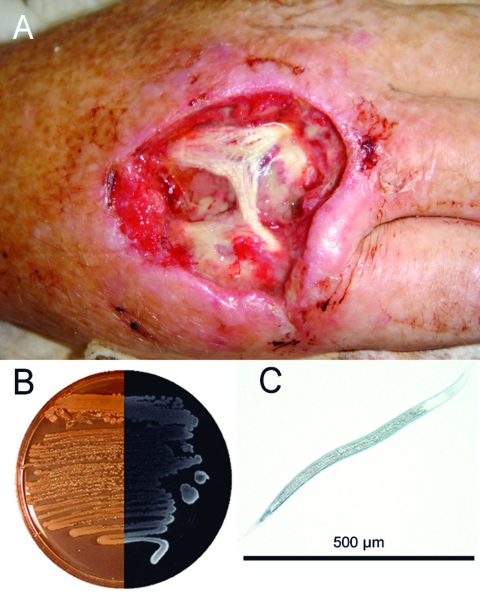
A) Hand of the patient infected with *Photorhabdus asymbiotica* after debridement. B) Composite photograph of a culture of *P. asymbiotica* taken in visible light and in darkness to demonstrate bioluminescence (Luria-Bertani medium). C) Soil nematode from which *P. asymbiotica* was isolated.

We report *Photorhabdus* infection in a 49-year-old Australian man who had fever and soft tissue infections of his right hand and left thigh in February 2006 (Figure 1). The patient had been digging fence post holes in the soft sandy soil at his house in Kingscliff (New South Wales), using his right hand as a scoop. The work caused some minor trauma to the skin of the dorsum of his hand. In the ensuing days, he experienced fever, and a severe local infection developed in his right hand. A secondary abscess developed in his left thigh ≈1 week later. *Photorhabdus* sp. was isolated in pure culture from pus collected from the patient's right hand. Blood cultures were negative for *Photorhabdus* sp.

The patient was initially treated with intravenous cephalosporins and his hand was subjected to debridement and reconstructive surgery. He was switched to a 5-week course of oral ciprofloxacin when the pathogen was identified, and he improved steadily.

We hypothesized that the *Photorhabdus* infection was transmitted by a previously unidentified insect-pathogenic nematode. To prove this hypothesis, seven 650-mL sandy soil samples were collected from the fence post holes dug by the patient and from the surrounding area. To each of these soil samples, 5 insect larvae (*Tenebrio mollitor*) were added as bait. Dead insects were removed from the containers 5 days later. Two of these insects were visibly luminescent.

*Photorhabdus* sp. was isolated from the luminescent insect hemolymph. Nematodes emerged from the insect cadavers within 14 days. These nematodes were surface sterilized, crushed individually in 100 μL Luria broth with a motorized mortar and pestle, and plated on Luria broth and NBTA agar (nutrient agar supplemented with bromothymol blue and triphenyltetrazolium chloride). Bacteria released from the intestine were bioluminescent and were confirmed to be *Photorhabdus* sp.

**Figure 2:**
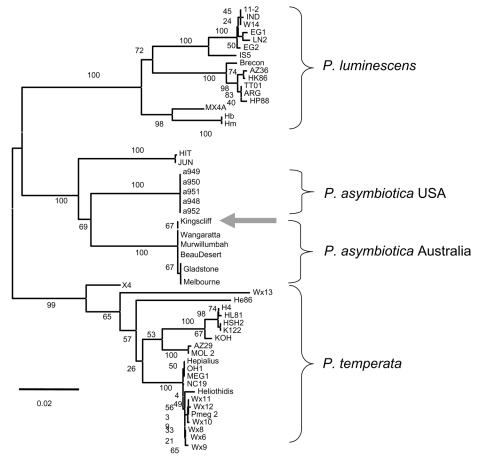
Phylogenetic tree of concatenated sequences of fragments of the *glnA* gene (474 bp) and the *gyrB* gene (576 bp) in 52 *Photorhabdus* isolates representing known diversity across the genus. The tree was constructed with the neighbor-joining algorithm and the K2-P method of distance estimation as implemented in MEGA version 3.0 (*12*). A total of 1,000 bootstrap replicates were performed, and the percentage of bootstrap trees supporting each node are given. The Kingscliff isolate (arrow) clusters with *P. asymbiotica* isolates from Australia, both in the concatenated tree (bootstrap score = 100%) and in individual gene trees (not shown). The scale bar shows percentage relatedness.

We tested whether the nematode-associated *Photorhabdus* from the infected insects was the same strain as that from the infected patient. The *Photorhabdus* isolates recovered from nematodes and from the patient's hand were compared on the basis of nucleotide sequences of 2 housekeeping genes, *glnA* and *gyrB*. The same gene fragments were also sequenced from a sample of 50 diverse *Photorhabdus* strains, including *P*. *asymbiotica* that had been isolated from patients in Australia and the United States. Multilocus sequencing is a powerful technique for typing and epidemiologic surveillance of many human pathogens (*10**,**11*). Phylogenetic analysis of these data confirmed that the human- and nematode-derived isolates of *Photorhabdus* were the same strain (referred to as *P*. *asymbiotica* Kingscliff). This strain clusters with other *P*. *asymbiotica* strains isolated from Australia (Figure 2).

Nematodes containing the Kingscliff bacteria were analyzed by amplifying the internal transcribed spacer region of crushed infective juvenile nematodes and of chromosomal DNA isolated from the nematodes (*13*). The PCR products were then purified and sequenced. BLAST (National Center for Biotechnology Information, Bethesda, MD, USA) searches showed that the Kingscliff nematode is a member of the genus *Heterorhabditis* and is most closely related to *H*. *indica* (98% identity) and to other members of this tropical group of isolates. Morphologic analysis and molecular analysis of coding DNA regions and mitochondrial DNA are currently underway.

Using the White-trap method (*14*) we have serially infected insects in vitro with the *P*. *asymbiotica* Kingscliff–*Heterorhabditis* complex, confirming that insects provide suitable prey. One feature of *Heterorhabditis* species is their specificity of association with their own species of bacterium. The 10 described nematode species do not grow and develop on bacteria from another species of nematode. Bacteria isolated from the human wound, the infected insect, and the nematode, as well as bacteria isolated from all 3 recognized species of *Photorhabdus* (*P*. *luminescens* TT01, *P*. *temperate* K122, and *P*. *asymbiotica* USA) and *Escherichia coli*, were tested with the Kingscliff nematode for growth in vitro on lipid agar media. Only Kingscliff bacteria from the wound, insect, and nematode supported growth and development of the Kingscliff nematode and the Kingscliff bacteria were retained by this nematode. In all other instances, infective juvenile nematodes failed to recover. *H*. *bacteriophora* nematodes failed to recover on Kingscliff bacteria from all 3 sources and on *Escherichia coli*. These data indicate that the Kingscliff bacterium is required for the growth and reproduction of the Kingscliff nematode, and lack of development or growth on any other strain indicates the specificity of this association, a characteristic of *Photorhabdus*-*Heterorhabditis* associations. *Photorhabdus asymbiotica* has been shown to be a nematode symbiont; the specific epithet is a misnomer.

## Conclusions

*P*. *asymbiotica* is not the first bacterial symbiont of nematodes to be associated with human disease. *Wolbachia*, an intracellular bacterial symbiont of the nematodes *Onchocerca volvulus* and *Brugia malayi*, has been implicated in the pathogenesis of 2 major human infectious diseases, river blindness and lymphatic filariasis (*15*). However, unlike *Wolbachia*, *P*. *asymbiotica* appears to actively reproduce in its human host. *O*. *volvulus* and *B*. *malayi* nematodes are borne by an insect vector. The insect-pathogenic nematode bearing *P*. *asymbiotica* does not appear to have been borne by an insect vector. Whether this nematode is able to penetrate intact human skin is unclear, although direct skin penetration by nematodes is well recognized (e.g., hookworm, *Strongyloides stercoralis*). Although the patient described here had a history of minor skin trauma, previous case reports suggest infection beginning in uninjured skin.

With continued population growth and movement, changes in human behavior, and changes in the environment, new human infectious diseases can be expected to continue to cross the species barrier. Given the dominance of invertebrate animal species in the biosphere, more invertebrate pathogens will likely emerge as agents of human infection.
